# Clinical Outcomes and Device-Related Complications Following Flow Diverter Stent Treatment of Unruptured Intracranial Aneurysms: A Single-Center Retrospective Study

**DOI:** 10.3390/diagnostics16172777

**Published:** 2026-08-29

**Authors:** Necat Islamoglu, Ali Can Yalcin, Ilker Coven

**Affiliations:** Konya City Hospital, 42020 Konya, Turkey; alican.yalcin1@saglik.gov.tr (A.C.Y.); ilker.coven@saglik.gov.tr (I.C.)

**Keywords:** intracranial aneurysm, flow diverter stent, nitinol-based stent, endovascular treatment, stent thrombosis, clinical outcomes, device-related complications, neurointervention

## Abstract

**Background/Objectives**: Flow diverter stents are widely used for the treatment of complex, unruptured intracranial aneurysms. Nitinol-based devices offer improved flexibility and vessel conformability; however, real-world clinical outcome data remain limited. This study aimed to evaluate the safety and clinical outcomes of nitinol-based flow diverter stents in a single-center cohort. We hypothesized that these devices would demonstrate high technical success rates, low rates of device-related complications, and favorable functional outcomes at 6 months. **Methods**: We retrospectively analyzed 109 patients with unruptured saccular intracranial aneurysms treated with nitinol-based flow diverter stents between 2020 and 2025. Patients with ruptured aneurysms, non-nitinol devices, or incomplete 6-month follow-up data were excluded. Technical success, device-related complications, and clinical outcomes were assessed using standardized imaging follow-up at 1 month (CT angiography) and 6 months (digital subtraction angiography). **Results**: Technical success was achieved in 109/109 cases (100%). The internal carotid artery cavernous segment was the most common aneurysm location. Intra- and peri-procedural device-related complications occurred in 12/109 (11%) patients, all of which were successfully managed without permanent neurological deficit. During the 6-month follow-up, post-procedural device-related complications occurred in 5/109 patients (4.6%), all due to delayed stent thrombosis requiring mechanical thrombectomy. One patient died due to persistent stent occlusion, and one patient developed unilateral lower-extremity plegia. Three patients experienced transient, non-device-related hypoesthesia that resolved during follow-up. At 6 months, most patients had favorable functional outcomes, with modified Rankin Scale scores of 0–1. Complete aneurysm occlusion was achieved in 83 of 108 patients (76.9%) at the 6-month angiographic follow-up. **Conclusions**: Flow diverter stents demonstrated high technical success and favorable clinical outcomes. Fishmouth deformity emerged as a potentially important technical factor associated with thrombotic complications and warrants careful recognition during deployment and follow-up.

## 1. Introduction

Intracranial aneurysms represent a significant neurovascular pathology with the potential for catastrophic subarachnoid hemorrhage if rupture occurs. Conventional endovascular treatments, such as coiling or stent-assisted coiling, have limitations in complex aneurysm configurations, particularly wide-necked, fusiform, or giant aneurysms, due to high rates of recanalization and retreatment [1,2].

Flow diverter stents (FDSs) have emerged over the past two decades as a paradigm-shifting endovascular technology that remodels the parent artery rather than treating the aneurysm sac exclusively. These devices are deployed across the aneurysm neck to alter intra-aneurysmal hemodynamics, promote thrombosis within the aneurysm, and facilitate endothelialization along the parent vessel, thereby achieving durable occlusion for aneurysms previously considered untreatable [3,4].

The adoption of flow diversion has expanded the range of aneurysms amenable to endovascular therapy, including large and giant unruptured aneurysms of the internal carotid artery and certain bifurcation aneurysms, with clinical studies demonstrating favorable occlusion rates and acceptable safety profiles [5,6,7,8]. Nevertheless, complications such as in-stent thrombosis, ischemic events, and perforator occlusion remain concerns that warrant careful procedural planning, optimal antiplatelet management, and vigilant follow-up [9,10,11,12].

Although multiple flow diverter devices have been developed, procedural success and long-term outcomes remain influenced not only by device design but also by deployment-related factors, including stent apposition and vessel conformability [13]. Mechanical deployment abnormalities, such as fishmouth deformity, may contribute to thrombotic complications through incomplete wall apposition and altered local hemodynamics. Therefore, understanding the clinical significance of such findings may improve procedural safety and patient outcomes.

Newer-generation nitinol-based flow diverters are designed to provide enhanced flexibility and conformability in the tortuous cerebrovascular anatomy. Given that a device’s material and design may influence its deployment behavior and wall apposition, evaluating these devices within a material-homogeneous cohort may facilitate a more focused assessment of device-related mechanical complications. Therefore, this study aimed to evaluate the safety, clinical and angiographic outcomes, and device-related complications of nitinol-based flow diverter stents in patients with unruptured intracranial aneurysms.

## 2. Materials and Methods

### 2.1. Study Design and Patient Selection

This retrospective single-center study was conducted at a tertiary neurointerventional center. Institutional Review Board approval was obtained, and the requirement for written informed consent was waived due to the study’s retrospective nature.

Between January 2020 and December 2025, a total of 149 consecutive patients treated with flow diverter stents for intracranial aneurysms were retrospectively reviewed. Patients were excluded from the analysis if they were treated with chromium–cobalt-based stents (*n* = 21), lacked 6-month follow-up imaging data (*n* = 10), or were treated for ruptured intracranial aneurysms (*n* = 9), as rupture-related clinical deterioration could confound outcome assessment. Patients treated with chromium–cobalt-based Pipeline flow diverter stents (*n* = 21) were excluded a priori to maintain a homogeneous device material cohort focused specifically on newer-generation nitinol-based flow diverters. Because differences in device material and design may influence mechanical properties, deployment behavior, vessel wall apposition, and conformability, inclusion of chromium–cobalt-based devices could have introduced device-related heterogeneity, particularly in the assessment of mechanical complications such as fishmouth configuration.

After applying the exclusion criteria, 109 patients with non-ruptured saccular intracranial aneurysms treated exclusively with nitinol-based flow diverter stents were included in the final analysis. All aneurysms included in the study were saccular in morphology. The aneurysm location was determined based on pre-procedural digital subtraction angiography and classified according to the arterial segment involved.

**Figure 1:**
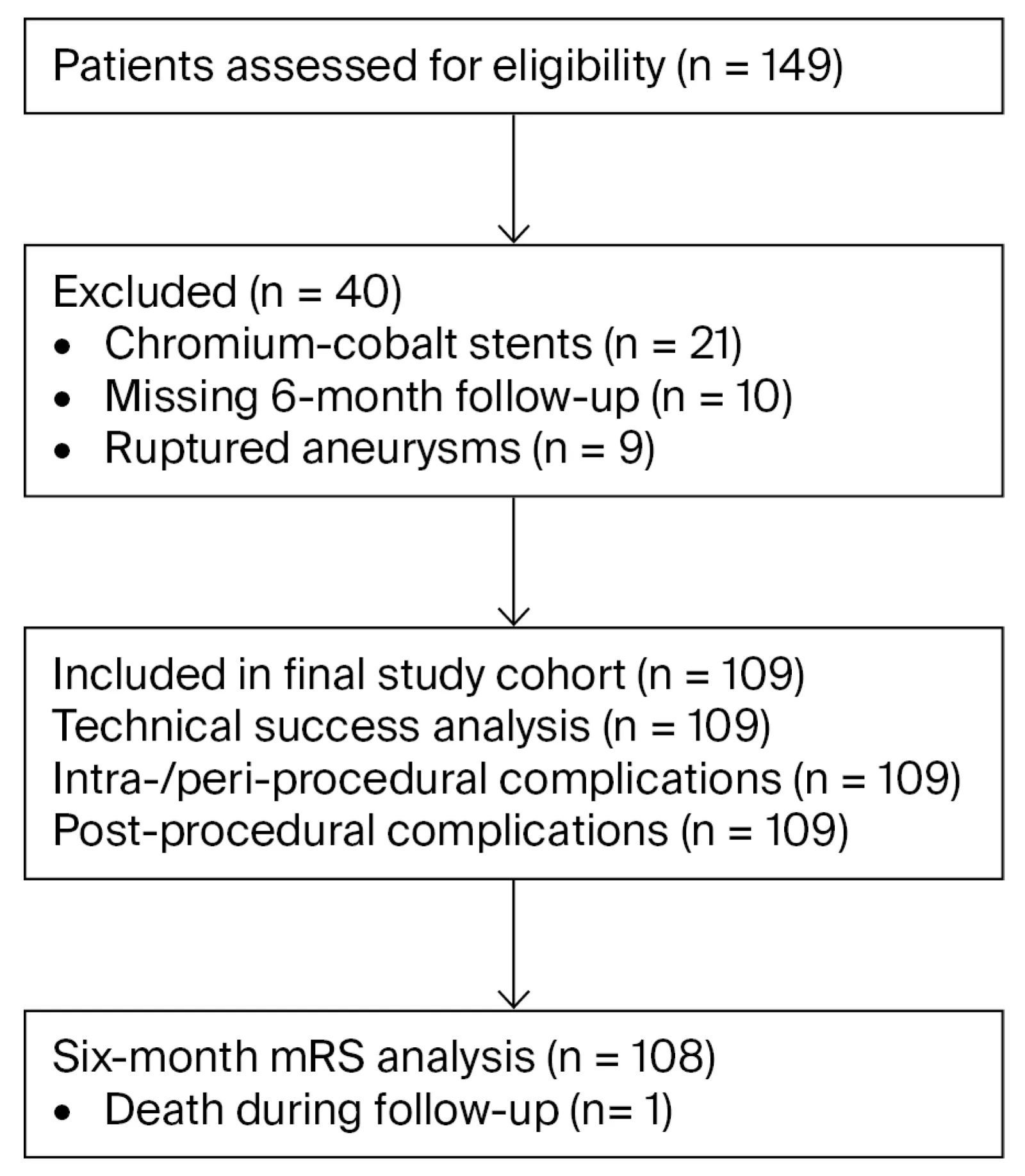
Patient flow diagram.

The exclusion criteria were applied sequentially. After each exclusion step, the remaining cohort was reassessed for eligibility. No patient met more than one exclusion criterion. The final study cohort of 109 patients constituted the denominator for analyses of technical success, intra-procedural complications, peri-procedural complications, and post-procedural device-related complications. Six-month modified Rankin Scale (mRS) outcomes were available for 108 patients; one patient died during follow-up due to ischemic stroke secondary to stent thrombosis and was therefore not included in the functional outcome assessment. A text-based patient flow is provided to ensure transparency of cohort selection and outcome-specific denominators (Figure 1).

### 2.2. Device Characteristics

Only nitinol-based flow diverter stents were used in this study. The devices implanted included Derivo Mini (*n* = 4), Derivo (*n* = 13), Silk Vista Baby (*n* = 17), and Silk (*n* = 75) flow diverter stents. Device selection was based on aneurysm location, parent vessel diameter, and operator preference.

### 2.3. Endovascular Procedure

All procedures were performed under general anesthesia by experienced neurointerventionalists. Patients received dual antiplatelet therapy (90 mg of ticagrelor twice daily and 100 mg of acetylsalicylic acid once daily) prior to the procedure according to the institutional protocol. Endovascular treatment was performed using a standard transfemoral approach. Flow diverter stents were deployed across the aneurysm neck, covering the parent artery segment.

Adjunctive coil use was retrospectively abstracted from procedural reports and angiographic records stored in the institution’s electronic medical records and PACS system. Coil use was recorded as a binary variable (yes/no) for each treated aneurysm. Aneurysm location in the adjunctive coiling subgroup was assigned using the same location classification procedure as applied to the overall study cohort. Location was determined based on pre-procedural digital subtraction angiography and classified according to the arterial segment involved. Analyses were performed at an aneurysm-based level. In patients with multiple aneurysms, each treated aneurysm was considered an independent analytical unit, and adjunctive coiling was attributed to the specific aneurysm treated during the index procedure. Adjunctive coiling was operationally defined as coil embolization performed in conjunction with flow diverter deployment during the same procedure. Adjunctive coiling was performed when one or more of the following criteria were present: (i) large or giant aneurysm size, (ii) high-flow inflow jet on pre- or intra-procedural angiography, (iii) persistent aneurysm filling after initial flow diverter deployment, or (iv) anticipated risk of delayed aneurysm thrombosis based on angiographic morphology. The final decision to perform adjunctive coiling was made at the operator’s discretion based on intra-procedural angiographic findings.

In anatomically suitable lesions, adjunctive coiling was used to accelerate aneurysm occlusion and reduce residual aneurysm filling, particularly when complete coil occlusion was not technically feasible or when aneurysm morphology suggested persistent inflow despite flow diverter deployment.

Peri-procedural antiplatelet management consisted of dual antiplatelet therapy with ticagrelor (90 mg twice daily) and acetylsalicylic acid (100 mg once daily), initiated prior to the procedure according to the institutional protocol. Following the procedure, dual antiplatelet therapy was continued for at least 6 months, after which patients were maintained on single antiplatelet therapy at the discretion of the treating physician.

Technical success was defined as successful delivery and complete deployment of the flow diverter stent at the intended target location with preservation of parent artery patency at the end of the procedure.

### 2.4. Follow-Up Protocol

All patients were followed up according to a standardized imaging protocol consisting of computed tomography angiography at 1 month and digital subtraction angiography 6 months following the procedure. Clinical and imaging outcomes included in the present analysis were based on the 6-month follow-up. In routine clinical practice, patients continue to undergo annual imaging surveillance at 1 year and up to 5 years thereafter.

Platelet function testing was not available at our institution during the study period and therefore was not performed routinely or as rescue testing following thrombotic events. Antiplatelet therapy compliance was assessed retrospectively through review of medical records, including outpatient follow-up notes and medication history. Discontinuation of antiplatelet therapy was defined as documented interruption or cessation of prescribed medication, whereas compliance was defined as continued use as documented in clinical records.

### 2.5. Outcome Measures

Primary outcome measures included the technical success rate and device-related complications during the 6-month follow-up period. Secondary outcome measures included clinical outcomes assessed using the modified Rankin Scale at 6 months and angiographic findings, including stent patency, deformation, or occlusion at the 6-month follow-up.

Complications were classified according to their temporal relationship to the procedure, in line with the Society of Interventional Radiology (SIR) Standards of Practice. Intra-procedural complications were defined as adverse events occurring during the intervention itself, prior to procedural completion. Peri-procedural complications were defined as events occurring immediately before, during, or within the early post-procedural period (up to 72 h). Post-procedural complications were defined as adverse events occurring after completion of the procedure during subsequent follow-up.

Device-related complications were evaluated, including in-stent thrombosis, distal vessel occlusion, in-stent stenosis, stent deformation, and fishmouth configuration. Fishmouth configuration was defined as an incomplete or asymmetric opening of the proximal or distal end of the flow diverter stent, resulting in a slit-like or V-shaped appearance on angiographic imaging, with incomplete apposition of the stent to the vessel wall. This configuration was assessed based on intra-procedural and follow-up angiographic findings.

Diffusion-weighted magnetic resonance imaging (DWI MRI) was not performed routinely in all patients. DWI MRI was selectively obtained in patients with suspected thromboembolic events, including those with angiographically confirmed in-stent thrombosis or new neurological symptoms during the peri- or post-procedural period. MRI was performed within 24–72 h following the thrombotic event or clinical suspicion.

MRI findings were recorded for outcome assessment. A lacunar infarct was defined as a focal diffusion-restricted lesion ≤15 mm in maximum diameter, located in the territory of a perforating artery, without associated cortical involvement. Infarct presence and characteristics were ascertained by review of formal radiology reports and confirmed by direct image review by the treating neurointerventional team.

Complications were analyzed at a patient-based level. Asymptomatic imaging findings without corresponding clinical symptoms were not classified as clinical complications. Neurological morbidity was defined as any new, persistent neurological deficit attributable to the procedure or device and present at hospital discharge or during follow-up. Complications were categorized according to timing as intra-procedural (occurring during the intervention) or peri-procedural (occurring within 72 h after the procedure). For subtype reporting, complication categories were defined as mutually exclusive at the patient level: fishmouth configuration only, in-stent thrombosis only, or combined fishmouth configuration with in-stent thrombosis.

Clinical outcomes were assessed using the modified Rankin Scale (mRS) at 6 months. Scores were obtained from outpatient clinic visits or, when unavailable, from structured review of medical records and were assessed by the treating neurointerventional team. Six-month mRS data were available for 108 of 109 patients; one patient died during follow-up and was therefore not included in the mRS distribution. No additional missing clinical outcome data were found.

Statistical analysis was performed using IBM SPSS Statistics for Macintosh, version 25.0 (IBM Corp., Armonk, NY, USA). Continuous variables are presented as the median and interquartile range (IQR), and categorical variables are expressed as frequencies and percentages. To evaluate the association between fishmouth configuration and intra- or peri-procedural in-stent thrombosis, patients were categorized according to the presence or absence of fishmouth configuration, and thrombosis rates were compared using Fisher’s exact test because of the small number of events. Odds ratios (ORs) were calculated to estimate the magnitude of the association. A two-sided *p*-value < 0.05 was considered statistically significant. Six-month angiographic outcomes and complication rates were also compared between patients treated with flow diverter stent placement alone and those treated with flow diverter stent placement with adjunctive coiling. Categorical outcomes were compared using Fisher’s exact test.

## 3. Results

A total of 109 patients with non-ruptured saccular intracranial aneurysms treated with nitinol-based flow diverter stents were included in the analysis. Baseline demographic and clinical characteristics are summarized in Table 1, and aneurysm morphological characteristics are presented in Table 2. All procedures were completed successfully, resulting in a technical success rate of 109/109 (100%).

Most aneurysms were small or medium in size, whereas giant aneurysms accounted for only four cases (3.7%). The median neck width was 5.1 mm, and the median dome-to-neck ratio was 1.7. Sidewall aneurysms predominated (77.1%), while bifurcation aneurysms accounted for 22.9% of cases. Posterior circulation aneurysms were uncommon (5.5%). Multiple flow diverter stents were required in 14 patients, and a total of 123 flow diverter devices were implanted in the study cohort (Table 2).

Aneurysms were most frequently located in the internal carotid artery, with the cavernous segment being the most common site, whereas the ACA A1 segment represented the least common location (Table 3).

Four different nitinol-based flow diverter stents were used in this cohort, with Silk stents constituting most implanted devices.

Adjunctive coil embolization, in addition to flow diverter stent placement, was performed in 49/109 patients (45.0%), including 32 cavernous ICA aneurysms (29.4%), 3 petrous ICA aneurysms (2.8%), 9 MCA M1 segment aneurysms (8.3%), and 5 ACA A2 segment aneurysms (4.6%). Regarding device type, adjunctive coiling was used in 39 of 75 Silk cases (52.0%), 4 of 17 Silk Vista Baby cases (23.5%), 5 of 13 Derivo cases (38.5%), and 1 of 4 Derivo Mini cases (25.0%). At the 6-month angiographic follow-up, complete aneurysm occlusion was observed in 45 of 49 patients (91.8%) treated with adjunctive coiling compared with 38 of 59 patients (64.4%) treated with flow diverter stent placement alone (*p* = 0.001). Intra- and peri-procedural complication rates were similar between the groups using adjunctive coiling and flow diverter stents alone (5/49 [10.2%] vs. 7/60 [11.7%], respectively; *p* = 1.000). Similarly, post-procedural device-related complications occurred in 2/49 patients (4.1%) in the adjunctive coiling group and 3/60 patients (5.0%) in the group using flow diverter stents alone (*p* = 1.000) (Table 4).

**Figure 2:**
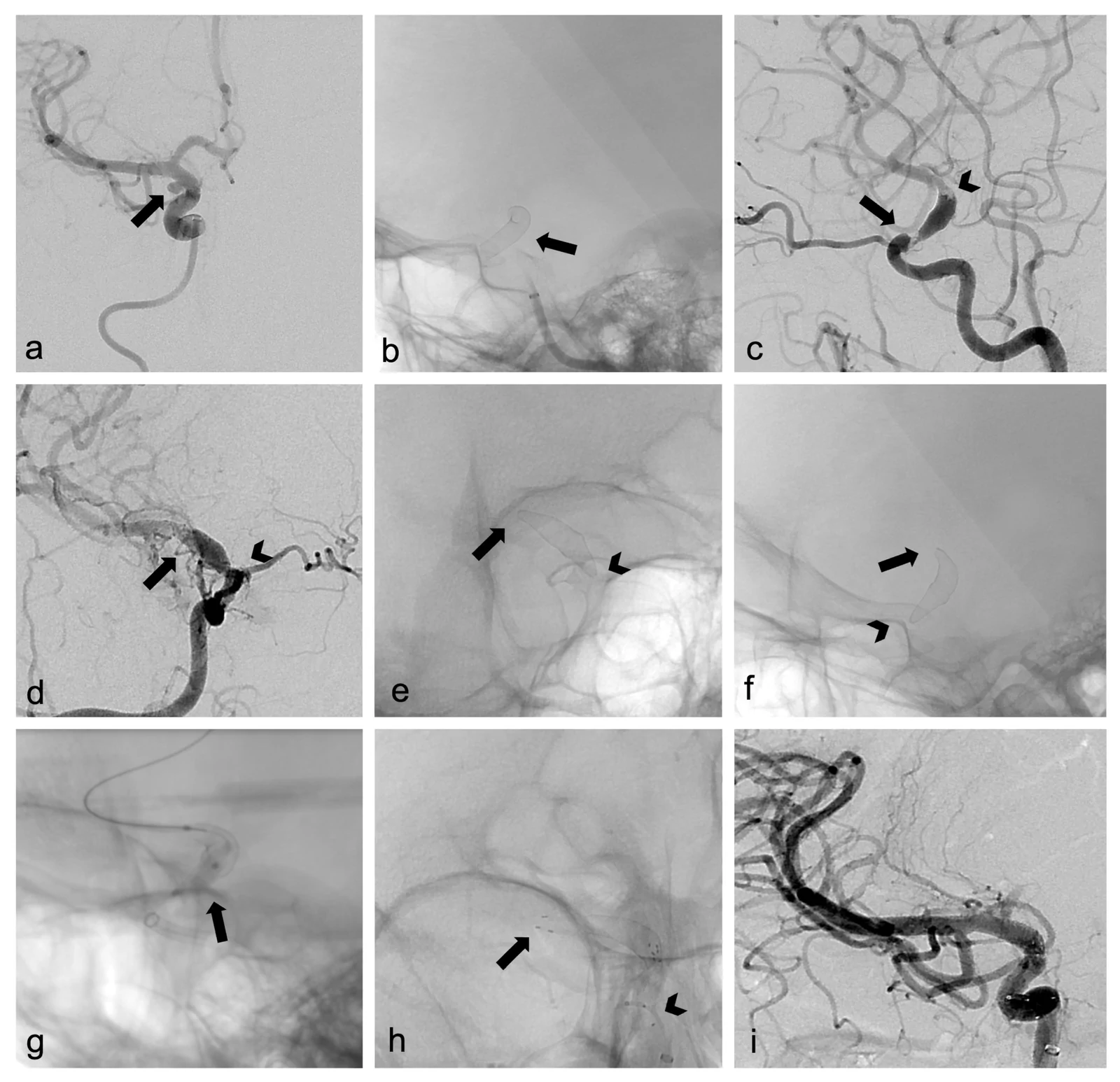
An aneurysm is demonstrated in the paraophthalmic segment of the right internal carotid artery (ICA) (**a**). A flow-diverting stent (Silk Vista, 4 × 15 mm) was deployed across the aneurysm neck (**b**). Following sudden onset of visual loss in the right eye on the day after the procedure, emergency angiography on the lateral projection demonstrated fishmouth deformity with associated thrombus at the proximal (arrow) and distal (arrowhead) ends of the stent (**c**). The same findings were demonstrated on the anteroposterior (AP) projection, showing proximal (arrowhead) and distal (arrow) fishmouth deformity (**d**,**e**), and were further confirmed on the lateral projection with proximal (arrowhead) and distal (arrow) involvement (**f**). Balloon angioplasty (Eclipse, 6 × 9 mm) was performed to correct the fishmouth deformity (**g**). Subsequently, telescopic stents (Atlas, 4 × 24 mm) were deployed in the distal (arrow) and proximal (arrowhead) segments (**h**). Follow-up angiography demonstrated complete stent patency and complete resolution of the fishmouth deformity (**i**).

Intra- and peri-procedural complications were identified in 12/109 patients (11%). Six patients had an isolated fishmouth configuration, three patients had a fishmouth configuration associated with in-stent thrombosis, and three patients had isolated in-stent thrombosis. In patients with a fishmouth configuration, the issue was successfully managed with percutaneous transluminal angioplasty (PTA) or via deployment of an additional telescoping stent. In one patient with an internal carotid artery paraophthalmic segment aneurysm, visual loss developed 15 h after treatment. Follow-up angiography demonstrated both proximal and distal fishmouth configurations with associated in-stent thrombosis, and the patient was treated with intra-arterial tirofiban, balloon angioplasty, and telescopic stent placement (Figure 2). The patient subsequently received hyperbaric oxygen therapy, resulting in complete resolution of visual symptoms. In cases with in-stent thrombosis, intra-arterial tirofiban was administered as a bolus dose (0.25–1.0 mg, according to angiographic thrombus burden), followed by an intravenous maintenance infusion at a dose of 0.1 μg/kg/min, leading to complete thrombus resolution in all patients. Post-procedural diffusion-weighted MRI was performed in patients with in-stent thrombosis, revealing a lacunar infarct in one patient without any associated clinical symptoms. No additional complications were observed in the remaining patients (Table 5). According to timing, seven complications occurred intra-procedurally, and five occurred during the peri-procedural period (within 72 h). In-stent thrombosis occurred in 3 of 9 patients (33.3%) with fishmouth configurations compared with 3 of 100 patients (3.0%) without fishmouth configurations. Fisher’s exact test demonstrated a statistically significant association between fishmouth configuration and intra- or peri-procedural in-stent thrombosis (OR = 16.17, *p* = 0.007).

During the 6-month follow-up period, device-related complications occurred in 5/109 patients (4.6%), all of which were stent thrombosis. These events involved stents placed in the internal carotid artery, basilar artery, and ACA A2 segment. All patients presented with acute ischemic stroke symptoms and underwent emergent mechanical thrombectomy (Table 6). None of the patients with post-procedural stent thrombosis (5/109, 4.6%) had a prior intra- or peri-procedural complication.

In one patient with basilar artery stent thrombosis, discontinuation of antiplatelet therapy was documented in clinical records, and the thrombosis was attributed to insufficient antiplatelet coverage. Mechanical thrombectomy was performed, resulting in restoration of stent patency, and the patient did not develop any permanent neurological deficits. In the remaining patients, compliance with antiplatelet therapy was confirmed based on review of clinical records. In two ICA cases and one ACA A2 case, distal stent fishmouth configuration was observed. Telescopic stent placement following successful thrombectomy restored complete vessel patency in two ICA cases. One patient with ICA stent thrombosis experienced persistent stent occlusion despite intervention and died due to a large ischemic stroke during intensive care follow-up. The patient with ACA A2 stent thrombosis demonstrated recurrent stent occlusion on follow-up imaging and developed unilateral lower-extremity plegia. Rescue platelet function testing was not performed in any of the five patients with delayed stent thrombosis because platelet function testing was not available at our institution during the study period.

Apart from these cases, no stent deformation, stenosis, or occlusion was detected at the 6-month angiographic follow-up in the remaining patients. Additionally, three patients developed transient, non-device-related hypoesthesia, which resolved completely during follow-up.

Six-month angiographic follow-up data were available for 108 of 109 patients. One patient died during follow-up due to ischemic stroke secondary to stent thrombosis and was therefore not available for angiographic outcome assessment. Angiographic occlusion outcomes are summarized in Table 7. Complete aneurysm occlusion was achieved in 83 of 108 patients (76.9%), whereas residual aneurysm filling was observed in 25 patients (23.1%). Additional angiographic classification data according to the Raymond–Roy and O’Kelly–Marotta grading systems are presented in Table 7.

At 6 months, clinical outcome data were available for 108/109 patients. Of these, 106/108 patients had an mRS score of 0, 1/108 had an mRS score of 1, and 1/108 had an mRS score of 3. One patient (1/109) died during follow-up due to stent thrombosis and was not included in the mRS distribution.

## 4. Discussion

In this single-center retrospective study, we evaluated the clinical outcomes and complications of nitinol-based flow diverter stents in the treatment of unruptured intracranial aneurysms. The principal findings of this cohort include a technical success rate of 100%, a relatively low incidence of device-related complications, and favorable functional outcomes at the 6-month follow-up.

The overall technical success rate observed in our series is consistent with previously published single-center and multicenter experiences using modern low-profile flow diverter devices [14,15,16]. Martínez-Galdámez et al. reported a technical success rate exceeding 90% in a multicenter cohort treated with the Silk Vista device, emphasizing the improved deliverability and wall apposition achieved with newer-generation nitinol stents [5,17]. Similarly, Vasconcellos de Oliveira Souza et al. demonstrated reliable deployment and favorable angiographic outcomes using Silk Vista Baby in distal aneurysms and those beyond the circle of Willis, highlighting the advantages of nitinol flexibility and conformability in tortuous cerebrovascular anatomy. Although our cohort predominantly consisted of proximal anterior circulation aneurysms, the complete technical success achieved across all cases reinforces the procedural reliability of nitinol-based flow diverters.

An additional finding of the present study was the higher 6-month complete aneurysm occlusion rate observed in patients treated with adjunctive coiling compared with those treated with flow diverter stent placement alone (91.8% vs. 64.4%, *p* = 0.001). Notably, this difference was not accompanied by an increase in either intra- and peri-procedural or post-procedural device-related complication rates. However, adjunctive coiling was not randomly assigned and was selected at the operator’s discretion based on aneurysm morphology and intra-procedural angiographic findings. Therefore, the observed association should not be interpreted as evidence of a causal benefit of adjunctive coiling. Prospective comparative studies are needed to determine whether adjunctive coiling provides an independent benefit in aneurysm occlusion following flow diversion.

In the present study, fishmouth configuration was significantly associated with intra- or peri-procedural in-stent thrombosis. In-stent thrombosis occurred in 3 of 9 patients (33.3%) with fishmouth configuration compared with 3 of 100 patients (3.0%) without fishmouth configuration (OR = 16.17, *p* = 0.007). Fishmouth deformity reflects incomplete stent apposition at the proximal or distal ends of the flow diverter and is often regarded as a technical deployment issue rather than a clinically relevant complication. Our findings suggest that such mechanical imperfections may be associated with an increased risk of thrombotic complications.

Although a statistically significant association was observed, a causal relationship cannot be established because of the retrospective study design and the small number of thrombotic events. Incomplete stent apposition may create local flow disturbances, areas of low wall shear stress, and subsequent platelet activation, mechanisms that are well recognized in the pathogenesis of early thrombus formation in intracranial stents. The coexistence of fishmouth deformity and stent thrombosis in a subset of patients in our cohort supports the hypothesis that mechanical factors, in addition to pharmacological antiplatelet efficacy, may play an important role in intra- and peri-procedural thrombotic events. Importantly, mechanical correction of fishmouth deformity using balloon angioplasty or telescopic stent placement resulted in restoration of adequate stent apposition and favorable angiographic outcomes [18].

Several anatomical and device-related factors may contribute to the development of fishmouth deformity following flow diverter deployment. These include vessel tapering; landing-zone diameter mismatch; excessive vessel tortuosity, particularly within the internal carotid artery siphon; and suboptimal sizing of the implanted device. Incomplete radial expansion may result in inadequate wall apposition, creating regions of disturbed flow and reduced wall shear stress that promote platelet activation and thrombus formation. Furthermore, incomplete apposition may impair endothelialization along the stent surface, potentially increasing the risk of delayed thrombotic events. These mechanisms provide a biologically plausible explanation for the association between fishmouth deformity and stent thrombosis observed in our cohort.

In the present cohort, no permanent neurological morbidity was observed as a result of intra- or peri-procedural events; however, a subset of patients required peri-procedural mechanical intervention due to technical deployment-related issues, most commonly fishmouth deformity. These findings indicate that, while peri-procedural complications in the strict clinical sense were not observed, peri-procedural technical problems requiring prompt correction were relatively common. When recognized early and managed with balloon angioplasty or telescopic stent placement, these issues did not result in adverse clinical outcomes.

During follow-up, device-related complications were infrequent and were exclusively related to delayed stent thrombosis, occurring in 5/109 patients (4.6%). This incidence is consistent with thromboembolic complication rates of approximately 3–6% reported in large flow diversion series. Although the overall incidence was low, the occurrence of persistent stent occlusion resulting in a fatal ischemic stroke underscores that delayed stent thrombosis remains the most clinically significant and potentially devastating adverse event associated with flow diversion.

Several factors may predispose to thrombotic complications following flow diverter implantation, including suboptimal stent apposition, vessel diameter mismatch, and inadequate antiplatelet therapy. Experimental and material-based studies have demonstrated that the mechanical properties of nitinol, such as radial force, size recovery, and wire composition, directly influence deployment behavior and apposition quality. Previous material-based studies have suggested that although nitinol-based flow diverters exhibit excellent size recovery and uniform deployment, they remain sensitive to vessel mismatch and deployment technique, potentially predisposing to malapposition-related thrombotic events if not carefully addressed. In line with these findings, angiographic review in our series revealed distal fishmouth deformity in several thrombotic cases, further supporting the role of mechanical and anatomical factors in delayed stent occlusion [19].

Antiplatelet therapy represents a critical component of flow diverter treatment. At our institution, dual antiplatelet therapy with ticagrelor and acetylsalicylic acid is routinely used in patients undergoing flow diverter implantation. Although ticagrelor provides direct and generally predictable P2Y12 inhibition, thrombotic events may still occur despite documented adherence to therapy. In the present cohort, discontinuation of antiplatelet therapy was documented in one of the five patients who developed delayed stent thrombosis, whereas adherence to the prescribed dual antiplatelet regimen was documented in the remaining four patients. However, platelet function testing was not available at our institution during the study period, and individual pharmacodynamic response to antiplatelet therapy could therefore not be objectively assessed. Consequently, inadequate platelet inhibition or pharmacological hyporesponsiveness cannot be excluded as a potential contributor to thrombotic events despite documented treatment adherence. Furthermore, rescue platelet function testing was not available in patients who developed delayed stent thrombosis. Therefore, despite documented adherence to dual antiplatelet therapy in four of the five patients with delayed thrombosis, pharmacological hyporesponsiveness cannot be excluded, and the relative contributions of mechanical factors, such as fishmouth configuration, and inadequate pharmacological platelet inhibition cannot be reliably distinguished in these cases. This limitation should be considered when interpreting the observed thrombotic complication rates.

Taken together, these findings suggest that fishmouth deformity should not be regarded as a benign imaging finding and may warrant early recognition and prompt mechanical correction, particularly when associated with angiographic flow abnormalities or thrombus formation. Prospective studies with comparative designs are needed to better define the true impact of fishmouth deformity on thromboembolic risk following flow diverter stent implantation. Although causality cannot be established from the present retrospective study, our observations suggest that fishmouth deformity may represent an underrecognized marker of thromboembolic risk following flow diverter implantation. The coexistence of fishmouth deformity and thrombotic events in several patients within our cohort highlights the potential clinical relevance of incomplete stent apposition. Early recognition and correction of fishmouth deformity may therefore help reduce the risk of subsequent ischemic complications. Future prospective studies are needed to validate this observation.

Importantly, all ruptured aneurysms were excluded from our analysis to avoid confounding effects on functional outcomes. This selection likely contributed to the favorable modified Rankin Scale scores observed at 6 months, with most patients achieving mRS scores of 0–1. Comparable functional outcomes have been reported in contemporary unruptured aneurysm flow diversion series, where strict patient selection and standardized antiplatelet regimens are employed [20]. The absence of major permanent neurological deficits in surviving patients further supports the safety of nitinol-based devices in this population.

This study has limitations inherent to its retrospective design and single-center nature. The standardized follow-up period of 6 months represents an additional limitation of this study. Although longer-term imaging follow-up was available for a subset of patients as part of routine clinical surveillance, follow-up durations beyond 6 months were heterogeneous and were therefore not included in the present analysis. Consequently, the study may not capture late-onset complications, including in-stent stenosis or delayed stent occlusion, and the durability of aneurysm occlusion beyond 6 months could not be systematically assessed. Furthermore, aneurysms with residual filling at 6 months may undergo progressive thrombosis and achieve complete occlusion during longer-term follow-up. Future studies with standardized long-term imaging follow-up are needed to assess the durability and evolution of these angiographic outcomes. Additionally, platelet function testing was not available at our institution, precluding objective assessment of antiplatelet responsiveness and limiting our ability to distinguish pharmacological hyporesponsiveness from mechanical contributors to thrombotic events. Nevertheless, the homogeneous device material, standardized follow-up protocol, and exclusion of ruptured aneurysms provide a focused evaluation of nitinol-based flow diverter performance in unruptured saccular aneurysms. In addition, adjunctive coiling was performed at the operator’s discretion rather than according to a randomized treatment strategy, introducing the possibility of selection bias and residual confounding in the subgroup comparison. Furthermore, the deliberate exclusion of chromium–cobalt-based pipeline devices improved material homogeneity but precluded direct comparison between nitinol- and chromium–cobalt-based flow diverters.

## 5. Conclusions

In this single-center retrospective study, treatment of unruptured saccular intracranial aneurysms with nitinol-based flow diverter stents was associated with high technical success and favorable clinical outcomes at the 6-month follow-up. Early intra- and peri-procedural device-related complications were infrequent and resolved without permanent neurological deficits, while delayed complications were primarily related to stent thrombosis. Overall, these findings support the safety and clinical utility of nitinol-based flow diverter stents in appropriately selected patients. Optimal device selection, proper stent deployment, and long-term antiplatelet management remain key factors for favorable outcomes. Early recognition of stent-related thrombosis or device deformation, such as fishmouthing, allows for timely intervention and may prevent permanent neurological sequelae.

## Figures and Tables

**Table 1:** Baseline characteristics.

Characteristic	Frequency
Age, median (IQR)	63 (51–70)
Female, *n* (%)	76 (69.7)
Hypertension, *n* (%)	70 (64.2)
Hyperlipidemia, *n* (%)	48 (44)
Diabetes mellitus, *n* (%)	65 (59.6)
Smoking, *n* (%)	81(74.3)
Baseline mRS (median, IQR)	0 (0–0)
Previous cerebrovascular event, *n* (%)	17 (15.6)

IQR: interquartile range; mRS: modified Rankin Scale.

**Table 2:** Aneurysm morphological characteristics (*n* = 109).

Characteristic	Value
Small (<7 mm), *n* (%)	32 (29.4)
Medium (7–10 mm), *n* (%)	41 (37.6)
Large (10–20 mm), *n* (%)	24 (22.0)
Very large (20–25 mm), *n* (%)	8 (7.3)
Giant (>25 mm), *n* (%)	4 (3.7)
Neck width, median, mm	5.1
Dome-to-neck ratio, median	1.7
Sidewall aneurysms, *n* (%)	84 (77.1)
Bifurcation aneurysms, *n* (%)	25 (22.9)
Posterior circulation aneurysms, *n* (%)	6 (5.5)
Multiple stent usage, *n* (%)	14 (12.8)
Total flow diverter stents implanted	123

**Table 3:** Aneurysm localization (*n* = 109).

Location	*n* (%)
ICA—cavernous segment	45 (41.3%)
ICA—petrous segment	7 (6.4%)
MCA—M1 segment	24 (22.0%)
MCA—M2 bifurcation	14 (12.8%)
ACA—A1 segment	2 (1.8%)
ACA—A2 segment	11 (10.1%)
Basilar artery	6 (5.5%)

ICA: internal carotid artery; MCA: middle cerebral artery; ACA: anterior cerebral artery.

**Table 4:** Comparison of clinical and angiographic outcomes between use of flow diverter stent alone and flow diverter stent with adjunctive coiling.

Outcome	FDS Alone	FDS + Adjunctive Coiling	*p*-Value
Complete aneurysm occlusion at 6 months	38/59 (64.4%)	45/49 (91.8%)	0.001
Residual aneurysm filling at 6 months	21/59 (35.6%)	4/49 (8.2%)	0.001
Intra-/peri-procedural complications	7/60 (11.7%)	5/49 (10.2%)	1.000
Post-procedural device-related complications	3/60 (5.0%)	2/49 (4.1%)	1.000

**Table 5:** Intra- and peri-procedural device-related complications and management.

Complication	Management	Outcome	Clinical Consequences	Stent Size (mm)	Vessel Caliber Discrepancy (mm)	Aneurysm Localization
DF + Clot	Tirofiban i.a. + PTA (Eclipse 6 × 9 mm)	Solved	None	3.5 × 25 Silk Vista	0.5	MCA M1
DF	PTA (4 × 7 mm Hyperform)	Solved	None	3 × 20 Silk Vista Baby	0.6	MCA M2 Bifurcation
PF + Clot	Tirofiban i.a. + Telescopic Stent (Atlas 3 × 21 mm)	Solved	None	3.5 × 20 Derivo	0.8	MCA M1
DF	PTA (Neurospeed 3 × 8 mm)	Solved	None	3 × 15 Derivo Mini	0.5	MCA M2 Bifurcation
PF	Telescopic Stent (Credo 5 × 20 mm)	Solved	None	4.5 × 30 Derivo	0.6	ICA Cavernous
PF + DF + Clot	Tirofiban i.a. +PTA (Eclipse 6 × 9 mm) + Telescopic Stent (Atlas 4 × 24 mm)	Solved	Transient Vision Loss	4 × 15 Silk	1	ICA Paraophthalmic
PF	Telescopic Stent (Leo Baby 2.5 × 18 mm)	Solved	None	2.75 × 15 Silk Vista Baby	0.4	ACA A2
PF	PTA (6 × 9 Eclipse)	Solved	None	4.5 × 20 Silk	0.7	ICA Paraophthalmic
PF	PTA (6 × 9 Eclipse)	Solved	None	4 × 20 Silk	0.8	ICA Petrous
Thrombotic in-stent occlusion	Tirofiban i.a.	Ipsilateral lacunar infarct	None	3.5 × 15 Silk	0.5	MCA M2 Bifurcation
Thrombotic in-stent occlusion	Tirofiban i.a.	Solved	None	4 × 15 Silk	0.6	ICA Cavernous
Thrombotic in-stent occlusion	Tirofiban i.a.	Solved	None	2.5 × 15 Silk Vista Baby	0.4	ACA A2

ICA: internal carotid artery; MCA: middle cerebral artery; ACA: anterior cerebral artery; DF: distal fishmouth; PF: proximal fishmouth; i.a.: intra-arterial. Fishmouth configuration refers to incomplete stent opening at the proximal (PF) or distal (DF) end. Cases labeled as PF + DF indicate simultaneous involvement of both ends. Each row represents a unique patient. Complication subtypes are reported as mutually exclusive categories; patients with combined fishmouth configuration and in-stent thrombosis are included only in the combined category and are not double-counted in individual subgroups.

**Table 6:** Postprocedural device-related complications (*n* = 5).

Complication	Management	Result	Clinical Outcome	Location
Stent thrombosis	Mechanical thrombectomy + telescopic stent	Recanalized	Mild hemihypoesthesia, dysarthria	ICA
Stent thrombosis	Mechanical thrombectomy + telescopic stent	Recanalized	No deficit	ICA
Stent thrombosis	Mechanical thrombectomy	Persistent occlusion	Death	ICA
Stent thrombosis	Mechanical thrombectomy	Recurrent occlusion	Lower-extremity plegia	ACA A2
Stent thrombosis (antiplatelet non-compliance)	Mechanical thrombectomy	Recanalized	No deficit	Basilar artery

**Table 7:** Six-month angiographic occlusion outcomes (*n* = 108).

Variable	*n* (%)
Complete aneurysm occlusion	83 (76.9)
Residual aneurysm filling	25 (23.1)
Raymond–Roy Grade I	83 (76.9)
Raymond–Roy Grade II	16 (14.8)
Raymond–Roy Grade III	9 (8.3)
O’Kelly–Marotta Grade A	5 (4.6)
O’Kelly–Marotta Grade B	8 (7.4)
O’Kelly–Marotta Grade C	12 (11.1)
O’Kelly–Marotta Grade D	83 (76.9)

## Data Availability

The data supporting the findings of this study are not publicly available due to privacy and ethical restrictions. De-identified data may be made available by the corresponding author upon reasonable request and subject to approval by the relevant institutional ethics committee.

## Abbreviations

The following abbreviations are used in this manuscript:

ACA	Anterior Cerebral Artery
CTA	Computed Tomography Angiography
DSA	Digital Subtraction Angiography
DWI	Diffusion-Weighted Imaging
FDS	Flow Diverter Stent
ICA	Internal Carotid Artery
IQR	Interquartile Range
MCA	Middle Cerebral Artery
MRI	Magnetic Resonance Imaging
mRS	modified Rankin Scale
OR	Odds Ratio
PACS	Picture Archiving and Communication System
PTA	Percutaneous Transluminal Angioplasty
